# eFEL: electrophysiology feature extraction library

**DOI:** 10.1093/bioinformatics/btag328

**Published:** 2026-05-22

**Authors:** Darshan Mandge, Anıl Tuncel, Aurélien Jaquier, Ilkan Kilic, Tanguy Damart, Henry Markram, Werner Van Geit, Rajnish Ranjan

**Affiliations:** Blue Brain Project, École Polytechnique Fédérale de Lausanne (EPFL), Campus Biotech, Geneva 1202, Switzerland; Blue Brain Project, École Polytechnique Fédérale de Lausanne (EPFL), Campus Biotech, Geneva 1202, Switzerland; Blue Brain Project, École Polytechnique Fédérale de Lausanne (EPFL), Campus Biotech, Geneva 1202, Switzerland; Blue Brain Project, École Polytechnique Fédérale de Lausanne (EPFL), Campus Biotech, Geneva 1202, Switzerland; Blue Brain Project, École Polytechnique Fédérale de Lausanne (EPFL), Campus Biotech, Geneva 1202, Switzerland; Blue Brain Project, École Polytechnique Fédérale de Lausanne (EPFL), Campus Biotech, Geneva 1202, Switzerland; Laboratory of Neural Microcircuitry, Brain Mind Institute, École Polytechnique Fédérale de Lausanne (EPFL), Lausanne 1015, Switzerland; Blue Brain Project, École Polytechnique Fédérale de Lausanne (EPFL), Campus Biotech, Geneva 1202, Switzerland; Blue Brain Project, École Polytechnique Fédérale de Lausanne (EPFL), Campus Biotech, Geneva 1202, Switzerland; Laboratory of Neural Microcircuitry, Brain Mind Institute, École Polytechnique Fédérale de Lausanne (EPFL), Lausanne 1015, Switzerland

## Abstract

**Motivation:**

Electrophysiological recordings are essential in experimental and computational neuroscience, providing insights into neuronal excitability and network behaviour. Extracting features such as action potential thresholds, widths, and firing patterns is conceptually straightforward, but in practice it is complicated by heterogeneous datasets and software environments, which hinder reproducibility and interoperability. A standardized, efficient, and portable framework is needed to ensure consistent analysis across platforms and alignment with community data standards.

**Results:**

We present the Electrophysiology Feature Extraction Library (eFEL), a cross-platform, open-source library that implements standardized definitions for over 90 electrophysiological features. eFEL combines a high-performance C++ core with a Python interface, supporting customizable feature dependencies, caching, and parallelization. It integrates with community standards such as Neurodata Without Borders and works seamlessly with common electrophysiology formats and simulation environments. Since its initial release in 2015, eFEL has been used in published studies spanning single-cell analysis, model optimization, multimodal fitting, and circuit simulations. eFEL provides a FAIR-compliant, versatile resource for reproducible electrophysiological data analysis.

**Availability and implementation:**

The eFEL library is publicly available at https://github.com/openbraininstitute/eFEL and the associated study data and scripts have been deposited in Zenodo at https://zenodo.org/records/17241835.

## 1 Introduction

Neurons communicate primarily through electrical signals. Action potentials generated by one neuron are transmitted to connected neurons through synaptic events, and neuronal information is believed to be represented both in the waveform of individual spikes and in the temporal patterns of their firing. Therefore, a detailed understanding of neuronal electrical behaviour is fundamental to neuroscience (Perkel and Bullock 1968, Softky and Koch 1993).

In experimental neuroscience, whole-cell patch-clamp electrophysiology remains the gold standard for directly recording cell’s electrical activity. In computational neuroscience, electrophysiological data provide the basis for mathematical models of neuron excitability and network behaviour. In both cases, quantitative feature extraction from recorded traces is essential, e.g. identifying action potentials and characterizing their amplitude, width, and firing frequency. These same features are required to validate and constrain computational models against experimental data.

Although the algorithms required to extract such features are relatively straightforward, practical challenges arise from the fragmented software ecosystem. At the time Electrophysiology Feature Extraction Library (eFEL) was developed, experiments were carried out mainly with software such as Igor Pro, pCLAMP, or MATLAB, while computational workflows typically relied on NEURON and C/C++. This heterogeneity hampered reproducibility and complicated the reuse of feature definitions across platforms. Therefore, a portable and standardized feature extraction library was needed, one that functions across operating systems and integrates with diverse software environments.

Feature extraction also involves subtleties. For example, reliable identification of action potential onset may depend on cell type: in some neurons, a fixed voltage threshold (e.g. +10 mV) is sufficient, whereas in others, a criterion based on the rate of voltage change (dV/dt) along with action potential amplitude is more appropriate. Dependent features, such as spike width, can only be measured accurately once action potential detection is robust and reliable. This underscores the need for a flexible framework in which feature dependencies can be defined and modified without altering the underlying code.

To address these challenges, we developed the eFEL, a cross-platform, customizable software library that provides standardized definitions and implementations for >90 electrophysiological features. eFEL supports both experimental and simulated data, including large-scale SONATA-based simulations (Dai *et al.* 2020). SONATA is a standardized file format for large-scale neuronal network simulations. It stores simulation outputs, including membrane voltage and spike time series, in a structured way that can be exchanged across simulation workflows. Because these outputs contain the same kinds of electrophysiological traces analysed in experiments, eFEL can be applied to SONATA simulation data in the same feature-based framework. eFEL integrates seamlessly with environments such as NEURON, MATLAB, Igor Pro, C/C++, and Python, and can analyse any dataset provided in a generic ASCII time-series format. In addition to current-clamp recordings, eFEL supports voltage-clamp data and extracellular microelectrode array (MEA) recordings.

At its core, eFEL is implemented in C++ for efficiency, with a Python interface that broadens accessibility and interoperability. eFEL has built-in support for open-community standard representations of the electrophysiology data, such as the Neurodata Without Borders (NWB) format (Teeters *et al.* 2015) and various other electrophysiology formats such as igor binary wave (IBW, Wavemetrics, Lake Oswego, OR, USA) and axon binary format (ABF, Molecular Devices, LLC.) via the Neo (Garcia *et al.* 2014) Python package. Both the NWB format and the Neo (Neuroscience Electrophysiology Object) package are endorsed by the International Neuroinformatics Coordinating Facility (INCF) (Abrams *et al.* 2022). The support for standard formats ensures interoperability with community standards, making it easier for researchers to integrate eFEL into their existing workflows. In recent years, the FAIR principles (Wilkinson *et al.* 2016)—Findability, Accessibility, Interoperability, and Reusability—have become a cornerstone in managing and disseminating scientific data. eFEL is designed to take advantage of FAIR-compliant data. With support for various electrophysiology data formats, researchers can seamlessly initiate their analysis using eFEL, facilitating a smoother transition and integration into their research workflow.

Typical use cases include extracting features across cell populations to identify distinguishing electrophysiological characteristics; performing multi-objective optimization (Druckmann *et al.* 2008) of the electrophysiological model (Markram *et al.* 2015, Van Geit *et al.* 2016, Iavarone *et al.* 2023, Reva *et al.* 2023); multimodal fitting of single-neuron models (Buccino *et al.* 2024) using intracellular and extracellular characteristics, constructing ion channel models by fitting voltage-clamp current features; synaptic feature fitting (Ecker *et al.* 2020) and feature extraction from circuit simulation data (Isbister *et al.* 2026). With its extensive feature set and broad compatibility, eFEL has become a versatile tool for both fundamental and applied neuroscience. The library is freely available via the Python Package Index (PyPI, https://pypi.org/project/efel) under the GNU Lesser General Public License Version 3.0.

## 2 Materials and methods

### 2.1 Dataset

The dataset used in this study was compiled from several publicly available sources, including Channelpedia (Ranjan *et al.* 2011), the Blue Brain Open Dataset (Ramaswamy *et al.* 2015), and the Single Cell dataset (Simko 2021). All data associated with this work have also been made available on the Zenodo data repository (https://zenodo.org/records/17241835).

### 2.2 Library overview

**Figure 1:**
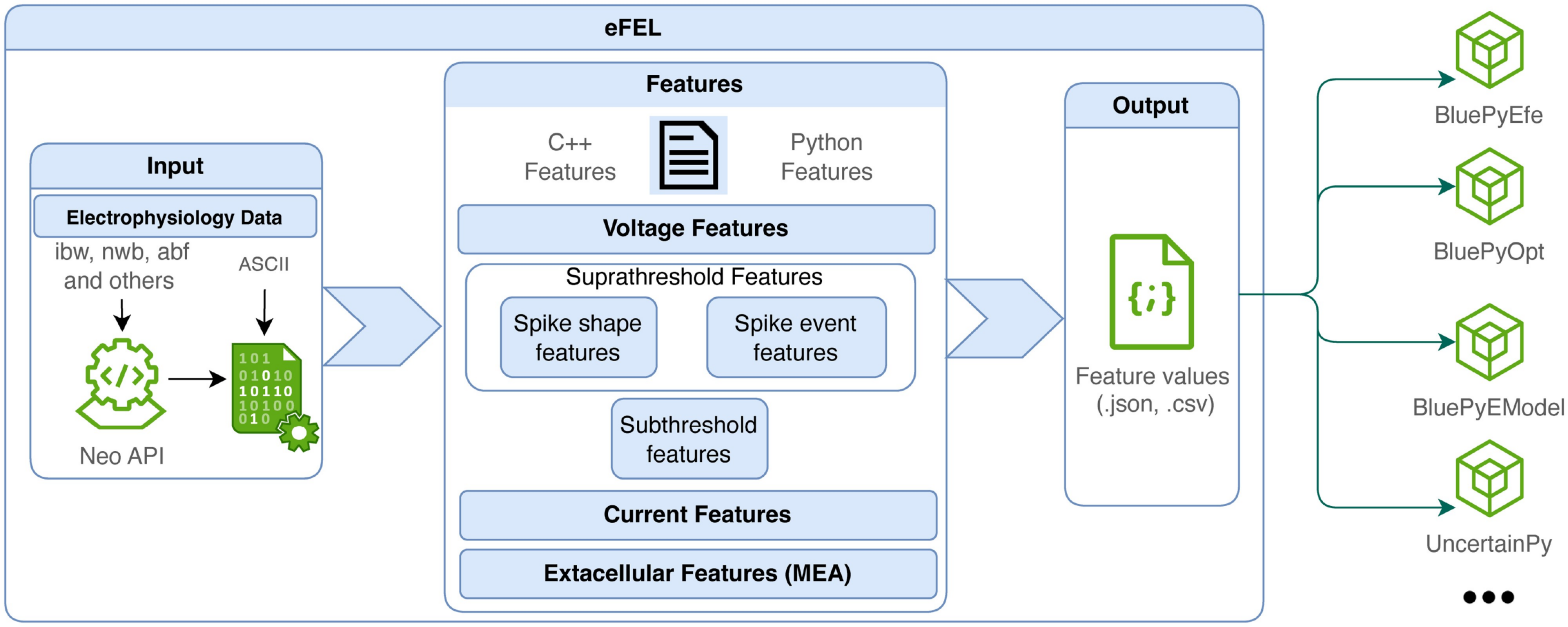
Overview of the eFEL library. The eFEL input module can accept multiple electrophysiology data formats that are translated to time series data. The electrical features written in C++/Python provide an output which can be saved as CSV or JSON formats and can be directly used as inputs to various software such as BluePyOpt and BluePyEModel.

The eFEL library focuses on the analysis of electrophysiological voltage and current traces. This section describes its design and its interaction with related software components (Fig. 1).

The eFEL application programming interface (API) accepts time-series data as input. Files can also be provided as input through the efel.io module, either as ASCII files or in electrophysiology formats supported by Neo (Garcia *et al.* 2014), such as Spike2, NeuroExplorer, AlphaOmega, and Axon. Feature computation can be customized with individual settings, such as the voltage threshold used for spike detection, interpolation step size, and related parameters. eFEL enables efficient parallel processing using the Python multiprocessing package as well as SCOOP (Hold-Geoffroy *et al*. 2014). The electrical features in eFEL are defined as C++ and Python features and fall into three categories: voltage features, current features, and extracellular features. These electrical features are described below. Finally, eFEL can save extracted features in both CSV and JSON formats. These outputs can be used directly in various software such as BluePyEfe (Rössert *et al.* 2024), BluePyOpt (Van Geit *et al.* 2016), and Uncertainpy (Tennøe *et al.* 2018).

### 2.3 Electrical features

**Figure 2:**
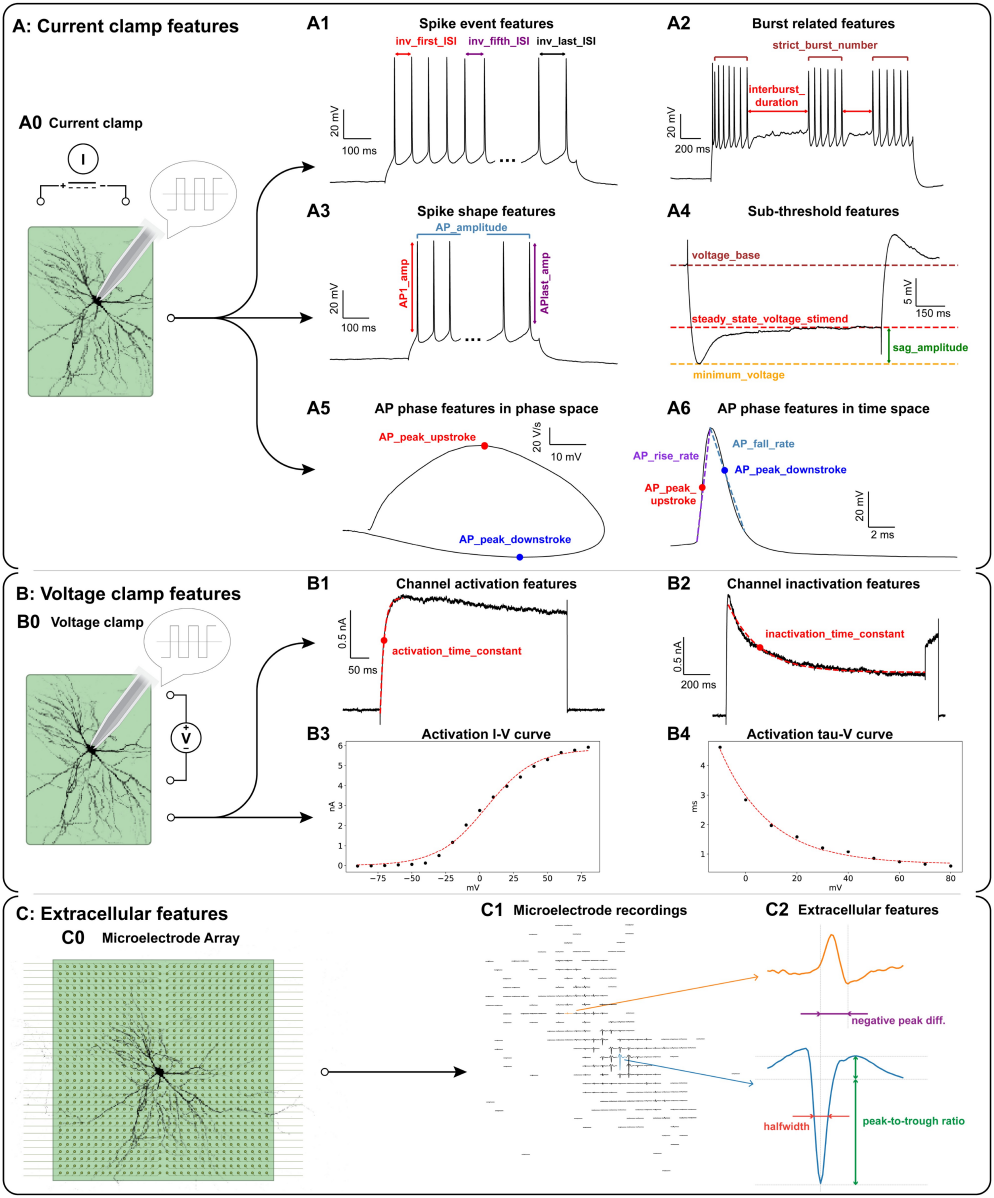
A schematic of eFEL features. (A) Voltage features extracted from current-clamp (A0) responses, inter-spike interval (ISI) timing features (A1), burst features (A2), AP amplitude features (A3), subthreshold features (A4), (V, dV/dt) phase space features shown on phase-space (A5), and time-space (A6). (B) Current features extracted from voltage-clamp (B0) responses, ion channel activation features (B1), inactivation features (B2), I–V curve of activation (B3), and activation time constant across input voltages. (C) Extracellular features (figure adapted from Buccino *et al*. 2024) extracted from microelectrode array (MEA) (C0), extracellular traces at multiple extracellular locations (C1), and selected traces displaying extracellular features (C2) shown on a larger timescale.

The eFEL library provides a selection of over 90 distinct features classified into the following types:

1. Voltage features
  - Spike event features (30 features, Fig. 2A1 and A2).
  - Spike shape features (36 features, Fig. 2A3).
    We have features for quantifying burst firing (Fig. 2A2), which are classified as Spike event features. The AP phase features are classified as Spike shape features (Fig. 2A5 and A6)
  - Subthreshold features (12 features, Fig. 2A4).
2. Current features (3 features, Fig. 2B)
3. Extracellular features (10 features, Fig. 2C)

**Figure 3:**
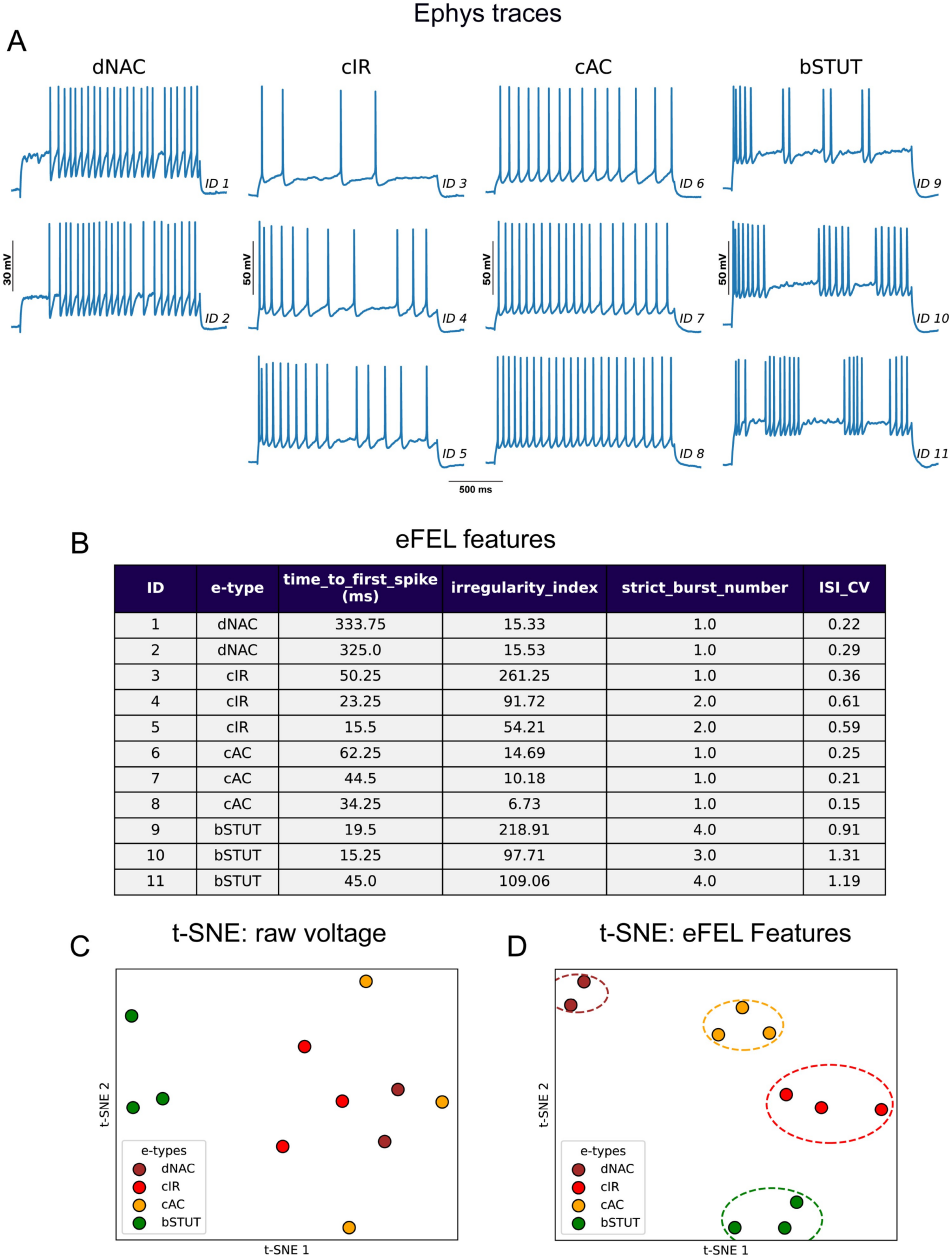
e-types analysis. (A) Voltage traces for four electrical types (e-types) of neurons from the rat somatosensory cortex (SSCx): delayed non-accommodating (dNAC), continuous irregular (cIR), continuous accommodating (cAC), and burst stuttering (bSTUT). Each e-type is presented in a separate column for direct comparison. (B) eFEL features extracted from the traces. (C) t-Distributed Stochastic Neighbour Embedding (t-SNE) plot of the raw voltage data, showing overlapping clusters. (D) Improved clustering of neuron e-types following feature extraction, as shown by the distinct clusters in the t-SNE plot.

**Figure 4:**
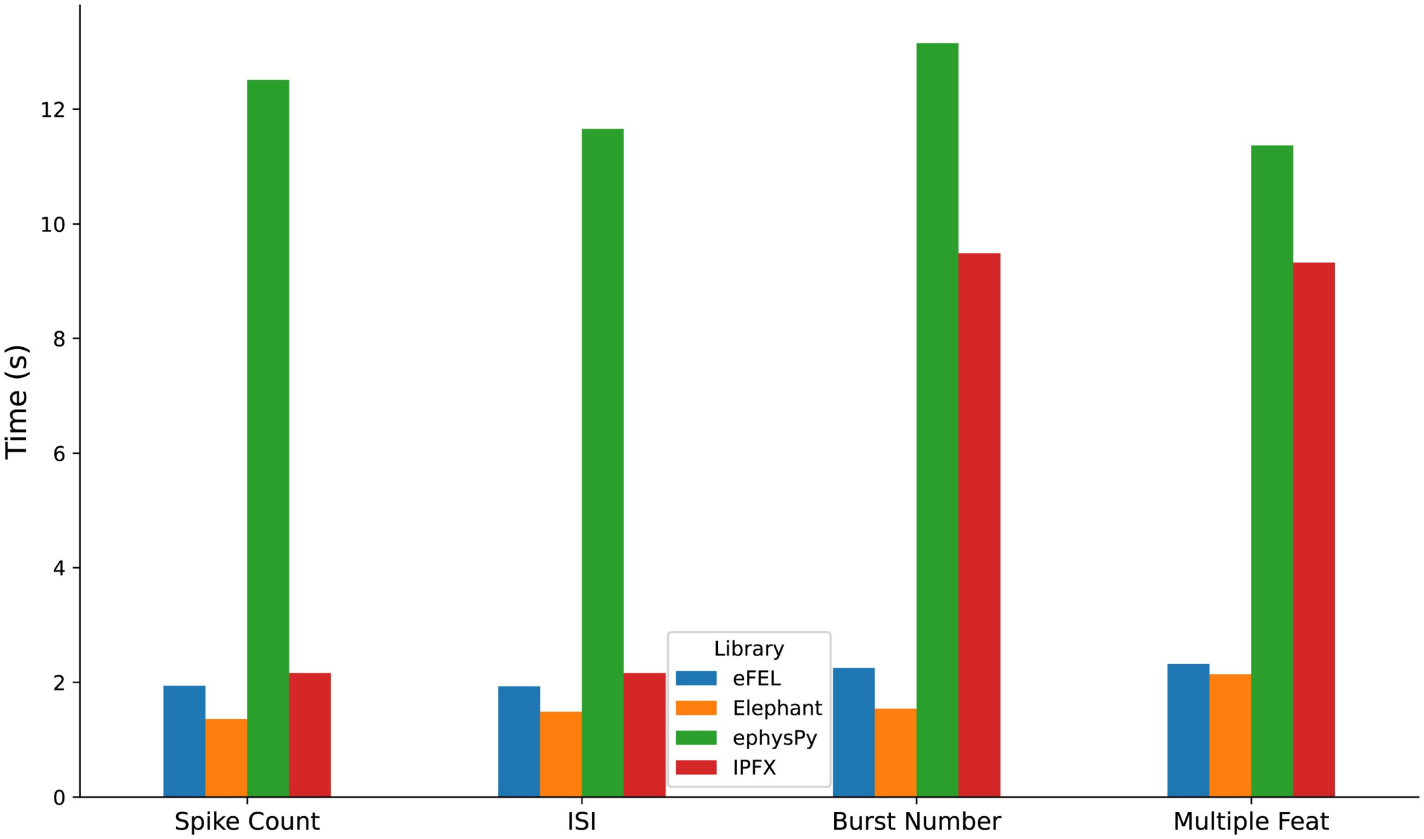
Benchmarking eFEL, Elephant, ephysPy, and IPFX. Total computation time for feature extraction on 800 traces for different features. The ‘Multiple Feat’ bars show the time for computing a set of features, namely Adaptation Index, Latency, inter-spike interval coefficient of variation (ISI CV), Mean ISI, Median ISI, First ISI, and Average Firing Rate.

Closely related features, e.g. AP1_peak and AP2_peak, were not counted separately. However, when these variants are included, the total number of features available in eFEL increases to 183. A complete list of the features is provided in the Tables 1 and 2, available as supplementary data at *Bioinformatics* online.

**Table 1:** Comparison of eFEL with related electrophysiology analysis libraries.^a^

Criterion	eFEL	Elephant	IPFX	ephysPy
**Implementation language**	C++ (with Python interface)	Python	Python	Python
**Approximate number of implemented features**	93 core features; 183 including variants	∼50	∼35 to 40	∼20 to 25
**AP and subthreshold feature granularity**	High: extensive AP event, AP shape, and subthreshold feature coverage	Low: not primarily designed for detailed intracellular waveform features	Moderate: standard AP, AHP, and passive membrane features	Basic to moderate: AP and passive metrics
**Primary data domain**	Primarily intracellular and selected extracellular/MEA features	Primarily extracellular; limited intracellular features	Intracellular	Intracellular
**Supported recording modalities**	In vitro and in silico intracellular current-clamp and voltage-clamp recordings; selected single-unit extracellular and MEA data	Spike trains, local field potentials (LFPs), and related extracellular/MEA analyses	In vitro whole-cell patch-clamp recordings	In vitro whole-cell patch-clamp recordings
**NWB support**	Yes, via Neo	Yes, via Neo	Yes	Yes, via AllenSDK
**Workflow integration**	Used in Open Brain Institute & EBRAINS workflows, including BluePyEfe, BluePyEModel, BluePyOpt, and Uncertainpy	Integrated with Neo/NeuralEnsemble workflows	Integrated with the Allen Cell Types pipeline	Python-based intracellular analysis workflows
**Initial release**	2015	2013	2017	2023
**Platform support**	macOS, Linux, and Windows	macOS, Linux, and Windows	macOS, Linux, and Windows	macOS, Linux, and Windows
**License**	LGPL-3.0	BSD-3-Clause	Allen Institute Software License (BSD-like with additional terms)	GPL-3.0
**Repository**	https://github.com/openbraininstitute/eFEL	https://github.com/NeuralEnsemble/elephant	https://github.com/AllenInstitute/ipfx	https://github.com/berenslab/ephyspy

a Libraries are compared based on implementation, approximate feature coverage, supported data modalities, interoperability, workflow integration, and platform support. Because these packages differ in scope and API design, feature counts are approximate and intended as a practical comparison rather than a strict one-to-one mapping.

Spike event features in the library use action potential (AP, or spike) detection when the voltage crosses a threshold, to extract several types of features from traces: spike count over the whole trace or part of it, mean firing frequency computation, burst detection and its related features (Fig. 2A2), such as the number of bursts, burst mean frequency, or inter-burst voltage, action potential timings, inter-spike intervals computation (Fig. 2A1), which can, in turn, be used by other features to determine the regularity or lack thereof of the spikes.

Spike shape features focus on the characteristics of the APs, such as the voltage values at the beginning and apex of the APs and between the APs, the amplitude of the AP (Fig. 2A3), the rate of voltage change during the rising and falling phases of the AP, the AP duration, the voltage of the first after hyperpolarization (AHP) and of the small after depolarization peak (ADP), and the slope of the (V, dv/dt) phase space of the rising phase of the APs (Fig. 2A5 and A6).

Subthreshold features are used on cell traces from subthreshold/hyperpolarized current injections. These include features such as the baseline voltage value, which reflects the resting membrane potential, the steady-state voltage during hyperpolarization, the ohmic input resistance, and various features describing the sag preceding steady-state when a hyperpolarizing stimulus is applied, such as the decay time constant describing exponential decay from voltage base to the sag, the time constant describing exponential from the sag to the steady state voltage, or the sag amplitude (Fig. 2A4).

Current features applied to voltage-clamp current traces allow the study of ion channel properties, including activation, inactivation, I-V and Tau-V plots (Fig. 2B).

Lastly, extracellular features can be computed on microelectrode array (MEA) data (Fig. 2C1) and include features specific to a channel of the MEA, such as peak-to-trough ratio or peak half-width duration (Fig. 2C2) and features relative to the multielectrode array channel with the largest amplitude, such as the positive or negative peak difference.

The eFEL library not only offers basic features but also extends to more sophisticated ones, such as the back-propagating action potential (BPAP) attenuation and AIS spike initiation detection. The BPAP feature operates with two voltage recordings and focuses on tracking the action potential from soma to its progression towards the dendrite. In this analysis, the baseline voltage from each voltage trace is first subtracted from the amplitude of their action potentials. These adjusted values are then used to calculate the attenuation, which is essentially the ratio of the action potential’s amplitude moving from the soma to the dendrite.

A comprehensive list of all eFEL features and their descriptions can be found in our detailed online documentation (https://efel.readthedocs.io/en/latest/).

### 2.4 Implementation details

#### 2.4.1 Interoperability with existing formats and software

The diversity of labs, tools, and data formats in neuroscientific research has historically posed challenges for data sharing and collaboration. Researchers often needed to convert data between various formats and adapt their software to new environments, which could divert attention from their main research goals. Recent efforts have led to improvements in interoperability within the field. Notable contributions to standardizing data formats have been made, which have streamlined some collaborative processes and reduced redundant tasks (Garcia *et al.* 2014, Teeters *et al.* 2015, Dai *et al.* 2020).

The eFEL library exemplifies these advancements by supporting standard data formats like NWB and those supported by the Neo API. It focuses on analysing electrophysiology data and can handle outputs from SONATA-based network simulations, enhancing its applicability across various studies. Additionally, data features extracted with eFEL are integrated into the Blue Brain Knowledge Graph, promoting better data discoverability and compliance with the FAIR principles (Findable, Accessible, Interoperable, and Reusable) as defined by the Neuroshapes community-driven models (González-Espinoza *et al.* 2023).

When analysing data from multiple cells to describe a cellular type, tools like BluePyEfe prove valuable by extracting and grouping features and computing statistical measures such as means and standard deviations. These grouped features can be utilized by BluePyOpt to optimize electrical models that mimic the observed characteristics, focusing on parameters like ion channel conductances and membrane properties (Van Geit *et al.* 2016, Rössert *et al.* 2024). This integration of software and data standards marks a positive shift towards more streamlined research practices, allowing scientists to dedicate more effort to core scientific enquiries.

#### 2.4.2 Extending Python with C++

In scientific computing, it is an established approach to blend interpreted languages, such as Python, with compiled languages like C, C++, and Rust. This strategy takes advantage of the performance benefits offered by compiled languages while preserving the flexibility and ease of use inherent in interpreted languages (Cottom 2003, Köster 2016, Harris *et al.* 2020). The eFEL library uses this strategy by creating Python extension modules in C++. These modules are integrated into the Python environment using the Extension module of the setuptools package, which is widely recognized for distributing Python packages. This integration means that installing eFEL is similar to installing any standard Python package, without requiring additional steps from the user.

#### 2.4.3 Feature dependency graph

Within eFEL, feature extraction can be conducted either on an individual basis or in groups. For the latter, eFEL creates a dependency graph that maps out the dependencies among features. In this graph, each feature is conceptualized as an algorithm, with nodes representing these algorithms and edges indicating their dependencies. This structured approach not only facilitates the efficient extraction of multiple features simultaneously but also provides the flexibility to substitute one feature implementation with another, such as for testing alternative approaches. A key element of eFEL’s efficiency is its use of memoization (Michie 1968, Norvig 1991). This technique involves storing results from dependent feature functions and retrieving these cached results for repeated inputs, thus avoiding redundant recalculations. Memoization effectively ‘remembers’ outcomes for specific sets of inputs, ensuring that subsequent calls with these inputs swiftly return the stored result rather than recalculating it. This approach substantially reduces the computational burden for repeated function calls with identical parameters.

#### 2.4.4 Gradual typing

Dynamically typed languages, favoured for rapid prototyping due to their development speed and convenience, often carry inherent risks of type-related errors that can complicate maintenance (Hanenberg *et al.* 2014, Ray *et al.* 2014, Gao *et al.* 2017). Gradual typing addresses these issues by allowing programmers to apply static or dynamic type checking within the same language through variable annotations that enable either compile-time or run-time error detection (Siek and Taha 2007).

This method has gained traction across the programming community, notably in dynamic languages through implementations like TypeScript, Flow (https://flow.org/), Hack (https://hacklang.org/), and RBS (https://github.com/ruby/rbs). These implementations integrate static type checking to combine the flexibility of dynamic typing with the robustness of static typing. Python’s community has adopted over 20 Python Enhancement Proposals focused on typing (https://peps.python.org/topic/typing/), which facilitates error detection and improves code maintenance by addressing common issues like the incorrect handling of null values and dynamic attribute initialization (Di Grazia and Pradel 2022, Khan *et al.* 2022).

In eFEL, gradual typing is particularly useful at the Python/C++ boundary. For example, trace fields such as T, V, stim_start, and stim_end must have predictable numeric types and shapes before being passed to the C++ core. Type annotations help detect mismatches early, improve IDE autocompletion for the extension API, and reduce errors caused by missing values or inconsistent return types.

## 3 Results

### 3.1 Using eFEL

Below is a simple example of using eFEL on voltage time series data from a current-clamp experiment. It can be found on the basic example page of the eFEL GitHub repository.

import efel

import numpy

# Load trace data from text file

data = numpy.loadtxt( ‘example_trace1.txt’ )

# Extract time (1 ^st^ column) and voltage (2 ^nd^ column)

time = data[:, 0]

voltage = data[:, 1]

# Initialize a dictionary to represent a trace

trace1 = {}

# Assign time and voltage arrays to the trace dictionary

trace1[ 'T' ] = time

trace1[ 'V' ] = voltage

# Define stimulus start/end times in ms

# Note: Values must be passed as single-element lists

trace1[ ‘stim_start’ ] = [700]

trace1[ ‘stim_end’ ] = [2700]

# Wrap the trace in a list, as eFEL e x pects a list of traces

traces = [trace1]

# Configure the spike detection threshold to -20 mV

efel.set_setting( ‘Threshold’ , -20)

# Calculate features AP_amplitude and voltage_base

traces_results = efel.get_feature_values(traces, [‘AP_amplitude’, ‘voltage_base’])

# traces_results returns a list of di c tionaries containing the

# calculated features

### 3.2 eFEL’s application to scientific data

eFEL not only serves as a tool for extracting features but can also be used to test hypotheses. Below are some possible scientific applications:

### 3.3 Classification of neuron firing types

Computational neuroscientists can use eFEL to classify the neuron firing types based on their intracellular patch-clamp recordings. In the following example, we used the electrophysiological data from rat P14 somatosensory cortex (SSCx) neurons (Markram *et al.* 2015, Ramaswamy *et al.* 2015). Figure 3A illustrates the results of feature extraction on the classification of four distinct neuron firing/electrical types (e-types): delayed non-accommodating (dNAC), continuous irregular (cIR), continuous accommodating (cAC), and burst stuttering (bSTUT). The dNAC traces (IDs 1 and 2) show delayed and consistent firing patterns characterized by a lack of accommodation, cIR traces (IDs 3, 4, and 5) exhibit varying firing irregularities, and the cAC traces (IDs 6, 7, and 8) demonstrate regular, consistent firing with a noticeable accommodation response, indicating a gradual reduction in firing rate over time. The bSTUT traces (IDs 9, 10, and 11) display a stuttering firing pattern, characterized by intermittent bursts of action potentials. This initial classification is based on manual observation of these features.

Figure 3B lists the features extracted from the traces. We selected specific features that would lead to effective clustering: time to first spike, irregularity index, strict burst number, and the coefficient of variation of the inter-spike interval (ISI CV). Time to first spike is useful for identifying dNAC neurons, which typically have a longer latency to their first spike. The irregularity index captures the high variability in the firing patterns of cIR neurons. A strict burst number quantifies the propensity for burst firing in bSTUT neurons. The ISI CV reflects the variability in spike timing, characteristic of cAC neurons, which show adaptive firing patterns.

The effectiveness of these selected features for neuron classification is illustrated in the t-SNE plots in Fig. 3C and D. Figure 3C shows a t-SNE plot of the raw voltage traces without using features, with overlapping clusters, indicating that the raw voltage traces alone do not fully capture distinctions among neuron e-types. In contrast, the t-SNE plot generated from eFEL-extracted features (Fig. 3D) shows distinct clusters, indicating that feature extraction significantly enhances their differentiation. t-SNE was performed separately using scikit-learn Python library after feature extraction and is not part of eFEL.

### 3.4 Voltage-clamp features

eFEL also supports feature extraction from currents recorded in voltage-clamp experiments, which are widely used to characterize ion channel kinetics. Supported features include maximum_current and kinetic time constants, such as activation and inactivation tau values, which can be used to derive I–V and tau–V relationships. Figure 2B illustrates feature extraction from voltage-clamp recordings obtained at 25°C from rat Kv1.1 channels expressed in Chinese hamster ovary (CHO) cells (Ranjan *et al.* 2019). A Jupyter notebook demonstrating this workflow is provided in the eFEL GitHub repository.

### 3.5 Phase-plane analysis features

Several features such as phaseslope_max, AP_phaseslope, AP_fall_rate, AP_fall_rate_change, AP_peak_downstroke, AP_peak_upstroke, AP_rise_rate and AP_rise_rate_change can be used for dynamic system and phase plane analysis of the intracellular data (Fig. 2A5 and A6). These features enable the analysis of data from biological experiments and simulations of multicompartmental and point-neuron (spiking neuron) models from a dynamical-systems perspective.

### 3.6 MEA recording extracellular features

eFEL can now be used to extract average e-features from MEA (multi-electrode array) recordings. These were used in a study using intracellular voltage recordings and extracellular MEA recordings to optimize an electrical model (Buccino *et al.* 2024). The following extracellular features are present in eFEL (Fig. 2C): peak_to_valley, halfwidth, peak_trough_ratio, repolarization_slope, recovery_slope, neg_peak_relative, pos_peak_relative, neg_peak_diff, pos_peak_diff, neg_image, pos_image. With the increasing use of HD-MEA (Müller *et al.* 2015) and Neuropixels probes (Steinmetz *et al.* 2021), the analysis of extracellular potentials is becoming increasingly important. eFEL provides both intracellular and extracellular features and can be an important tool for future neuroscientists studying such multimodal systems.

### 3.7 Benchmark and comparison

To benchmark eFEL’s (v 5.7.10) computational efficiency and feature set coverage, we compared it against three commonly used electrophysiological libraries: Elephant (1.1.0) (Denker *et al.* 2024), ephysPy (0.0.5) (https://github.com/berenslab/ephyspy), and IPFX (2.0.0) (https://github.com/AllenInstitute/ipfx). The analysed features included Spike Count, Inter-Spike Interval (ISI), and Burst Number. Additionally, we evaluated each library’s ability to compute a set of commonly used features (Adaptation Index, Latency, ISI CV, Mean ISI, Median ISI, First ISI, and Average Firing Rate), allowing for a more comprehensive assessment of feature extraction capabilities.

The benchmarking tests were conducted using 800 traces recorded from mouse thalamic cells (Iavarone *et al.* 2023). To ensure robustness, each feature extraction was repeated 10 times, and computation times were averaged and converted to seconds (s) for standardized comparison across libraries. All benchmarking computations were performed on a single node of a high-performance computing (HPC) cluster, with an allocation of 2 CPUs [Intel(R) Xeon(R) Gold 6248 CPU @ 2.50 GHz, with a maximum clock speed of 3.2 GHz] and 4 GB of memory. The results are summarized in Fig. 4. To complement the runtime benchmark, Table 1 summarizes key differences among these libraries with respect to feature coverage, supported data modalities, software interoperability, and use with intracellular and extracellular recordings.

While Elephant demonstrated superior speed, it does not offer the same extensive feature set that eFEL provides. IPFX delivers competitive performance for individual feature computations but tends to slow down with multiple feature extractions. In contrast, eFEL is optimized for handling multiple features by efficiently reusing intermediary results through its dependency structure. Although this structure introduces some overhead, it allows for greater customization in feature extraction. Combined with its broader feature set, eFEL offers a versatile and powerful tool for analysing diverse neuronal data.

### 3.8 Customizing eFEL

Users can customize eFEL to add new features using Python or C++ functions. In this section, we explain how to add a new Python feature in eFEL. Let’s consider adding a feature called ‘spike_count’ to find the number of spikes in a trace. This feature already exists in eFEL but is a useful example for understanding how to add new e-features.

1. Clone the code from the eFEL GitHub repository.

2. Create a Python function for the feature. Implement the feature in the efel/pyfeatures/pyfeatures.py file. Your function should take the necessary trace data as input and return the calculated feature value as below

def spike_count() -> np.ndarray:

   “““Get spike count.”““

   peak_indices = get_cpp_feature(“peak_indices”)

   ifpeak_indices isNone:

    returnnp.array([0])

return np.array([peak_indices.size])

The spike_count() function operates as follows:

- It calls the get_cpp_feature() function to obtain the indices of voltage peaks, which correspond to spike occurrences. get_cpp_feature() is a built-in eFEL function used to access features implemented in C++.
- If no peaks are detected (peak indices is None), it returns an array containing a single zero, indicating no spikes.
- Otherwise, it returns an array with a single element equal to the size of peak_indices, which represents the total number of spikes.
- The function returns a NumPy array to maintain consistency with other feature extraction functions.

3. Register the new feature. In the same pyfeatures.py file, add your new feature to the allfeatures dictionary:

allfeatures = {

   # … existing features …

   ‘my_new_feature’: spike_count,

}

4. Use the new feature. You can now use it like any other eFEL feature:

import efel

# … set up trace dictionary …

feature_values= efel.get_feature_values([trace], [ ‘spike_count’ ])

### 3.9 Cross-platform compatibility

eFEL works across all the major platforms such as macOS, Ubuntu and Windows for x86-64 and AMD64-based systems. eFEL results are reproducible across all these platforms. Each eFEL release includes Python wheels for versions 3.9–3.12 for macOS 12, Ubuntu 22.04, and current Windows systems.

## 4 Discussion

eFEL is a comprehensive electrophysiological feature-extraction tool designed to extract features from time-series data recorded from neurons (both in vitro and in silico). This paper introduces the eFEL library, benchmarks the library alongside other software in the field, demonstrates the ease of adding new features, and provides several published use cases. The use of eFEL is not limited to extracting features of electrical recordings. eFEL can be used to obtain corresponding features from any time series data. However, eFEL is not a spike sorting (Quiroga 2012) software, which involves identifying and separating action potentials from individual neurons in extracellular recordings. The extracellular features included in eFEL are mainly used for extracting average features from multiple traces of MEA data.

The library supports feature definitions in Python. Python has become the programming language of choice for neuroscientists, with most neuroscience software packages written in this language. This can encourage neuroscientists to contribute new features to eFEL.

### 4.1 eFEL-enabled studies

Several studies and software using eFEL have been published by the EPFL Blue Brain Project (BBP) and researchers worldwide using the library to extract e-features from experimental and model recordings. Some of the Blue Brain Project studies are listed below:

BluePyOpt and BluePyEModel (Mandge *et al.* 2023) use e-feature-based score metrics (Druckmann *et al.* 2007) extracted using eFEL for multi-objective model optimizations. The approach leads to more accurate models and provides a clear measure of model quality (Schürmann *et al.* 2022). Several studies have used it for constructing single neuron models of various brain regions (Markram *et al.* 2015, Arnaudon *et al.* 2023, Reva *et al.* 2023, Isbister *et al.* 2026) for rat somatosensory cortex, for thalamoreticular circuit (Iavarone *et al.* 2023), and for rat hippocampus (Romani *et al.* 2024). eFEL features scores as objectives have also been used to generalize neuron models for various morphological types (Arnaudon *et al.* 2023, Reva *et al.* 2023).

Neuron modelling studies have used eFEL features to validate the emergent properties such as somatic firing, backpropagating action potential (bAP), dendritic excitatory postsynaptic potential (EPSP) validation (Ecker *et al.* 2020, Reva *et al.* 2023). Such model validations are also implemented in the BluePyEModel Python package and PSP-validation (Post-Synaptic Potential Validation) Python packages.

The study by (Buccino *et al.* 2024) used extracellular features which have also been implemented in eFEL. Extracellular recordings using high-density microelectrode array (HD-MEA) and intracellular patch-clamp recording features were used to fit single neuron models.

Popular Python packages, such as BluePyOpt, BluePyEfe, BluePyEModel, and NeuroFeatureExtract (Bologna *et al.* 2021), utilize the eFEL library for feature extraction. A Google Scholar search using the keywords ‘Electrophysiology Feature Extraction Library eFEL’ returns >35 citations.

As summarized in Table 1, eFEL is distinguished by the breadth of its intracellular feature set, its support for current-clamp, voltage-clamp and selected extracellular/MEA analyses, and its integration with interoperable workflows spanning both experimental and simulation data. These differences reflect a design emphasis on reusable electrophysiology feature extraction across multiple modalities and software environments.

The widespread use of eFEL, together with its open-source development model, has supported progress in neuroscience. As the field continues to evolve, eFEL’s adaptability positions it as a useful resource for future neurophysiological research.

## Supplementary Material

btag328_Supplementary_Data
