# Supplementary material for: eFEL: electrophysiology feature extraction library
Source: Bioinformatics. 2026 May 22;42(6):btag328. doi: 10.1093/bioinformatics/btag328 (PMC13257855; doi:10.1093/bioinformatics/btag328)
Supplement: btag328_Supplementary_Data [file btag328_supplementary_data.docx]

Supplementary table 1: Voltage features

| **Spike event features** | **Spike shape features** | **Subthreshold features** |
| --- | --- | --- |
| time_to_first_spike,  time_to_second_spike,  time_to_last_spike | peak_voltage | steady_state_voltage_stimend,  steady_state_hyper,  steady_state_voltage,  voltage_base,  voltage_after_stim,  steady_state_current_stimend |
| inv_time_to_first_spike | AP_amplitude | current_base |
| ISI_values,  doublet_ISI,  all_ISI_values | mean_AP_amplitude,  AP_height,  AP1_peak,  AP2_peak | time_constant,  decay_time_constant_after_stim,  multiple_decay_time_constant_after_stim |
| inv_first_ISI,  inv_second_ISI,  inv_third_ISI,  inv_fourth_ISI,  inv_fifth_ISI,  inv_last_ISI,  inv_ISI_values | AP_Amplitude_change | sag_time_constant |
| spike_count,  spike_count_stimint,  number_initial_spikes | AP_amplitude_from_voltagebase, AP2_AP1_diff,  AP2_AP1_peak_diff,  amp_drop_first_second,  amp_drop_first_last,  amp_drop_second_last,  AP_amplitude_diff | sag_amplitude |
| mean_frequency | max_amp_difference | sag_ratio1 |
| ISI_semilog_slope | min_AHP_values,  min_AHP_indices | sag_ratio2 |
| ISI_log_slope | AHP_depth_slow, AHP_depth_abs,  AHP_depth_abs_slow | ohmic_input_resistance,  ohmic_input_resistance_vb_ssse |
| ISI_CV,  ISI_log_slope_skip | AHP_slow_time,  AHP_depth | voltage_deflection_vb_ssse,  voltage_deflection,  voltage_deflection_begin |
| irregularity_index | AHP_depth_diff | minimum_voltage |
| adaptation_index,  adaptation_index_2 | fast_AHP | maximum_voltage |
| burst_mean_freq,  strict_burst_mean_freq,  burst_begin_indices,  burst_end_indices,  burst_ISI_indices | fast_AHP_change | maximum_voltage_from_voltagebase |
| burst_number,  strict_burst_number | AHP_depth_from_peak |  |

Supplementary table 1: Voltage features (contd.)

| **Spike event features** | **Spike shape features** | **Subthreshold features** |
| --- | --- | --- |
| interburst_voltage,  strict_interburst_voltage | AHP_time_from_peak, AHP1_depth_from_peak,  AHP2_depth_from_peak |  |
| interburst_min_values,  interburst_min_indices | ADP_peak_values,  ADP_peak_indices |  |
| postburst_min_values,  postburst_min_indices | ADP_peak_amplitude |  |
| time_to_interburst_min | depolarized_base |  |
| single_burst_ratio | min_voltage_between_spikes,  min_between_peaks_values,  min_between_peaks_indices |  |
| spikes_per_burst,  spikes_in_burst1_burst2_diff,  spikes_in_burst1_burstlast_diff | AP_duration_half_width_change,  AP_duration_change,  AP2_AP1_begin_width_diff |  |
| spikes_per_burst_diff | AP_duration_half_width,  AP_duration,  AP_width,  AP_width_between_threshold,  spike_half_width,  AP1_width,  AP2_width,  APlast_width,  spike_width2,  AP_begin_width,  AP1_begin_width,  AP2_begin_width,  AP_rise_indices,  AP_fall_indices |  |
| peak_time peak_indices | AP_begin_voltage, AP1_begin_voltage,  AP2_begin_voltage |  |
| interburst_duration | AP_begin_time, AP_begin_indices,  AP_end_indices |  |
| interburst_15_percent_values,  interburst_20_percent_values,  interburst_25_percent_values,  interburst_30_percent_values,  interburst_40_percent_values,  interburst_60_percent_values,  interburst_15percent_indices,  interburst_20percent_indices,  interburst_25percent_indices,  interburst_30percent_indices,  interburst_40percent_indices,  interburst_60percent_indices | AP_peak_upstroke |  |
| time_to_postburst_slow_ahp | AP_peak_downstroke |  |

Supplementary table 1: Voltage features (contd.)

| **Spike event features** | **Spike shape features** | **Subthreshold features** |
| --- | --- | --- |
| postburst_slow_ahp_values,  postburst_slow_ahp_indices | AP_rise_time |  |
| postburst_fast_ahp_values,  postburst_fast_ahp_indices | AP_fall_time |  |
| postburst_adp_peak_values,  postburst_adp_peak_indices | AP_rise_rate |  |
| time_to_postburst_fast_ahp | AP_fall_rate |  |
| time_to_postburst_adp_peak | AP_rise_rate_change |  |
| check_ais_initiation | AP_fall_rate_change |  |
|  | AP_phaseslope |  |
|  | phaseslope_max |  |
|  | initburst_sahp |  |
|  | initburst_sahp_ssse |  |
|  | initburst_sahp_vb |  |
|  | bpap_attenuation |  |

Supplementary table 2: Voltage-clamp, Extracellular and Other features

| **Voltage-clamp features** | **Extracellular features** | **Other features** |
| --- | --- | --- |
| activation_time_constant | peak_to_valley | depol_block_bool |
| deactivation_time_constant | halfwidth | impedance |
| inactivation_time_constant | repolarization_slope |  |
|  | recovery_slope |  |
|  | neg_peak_relative |  |
|  | pos_peak_relative |  |
|  | neg_peak_diff |  |
|  | pos_peak_diff |  |
|  | neg_image |  |
|  | pos_image |  |
